# DeepTDM: Deep Learning-Based Prediction of Sequential Therapeutic Drug Monitoring Levels of Vancomycin

**DOI:** 10.1109/JTEHM.2025.3623605

**Published:** 2025-10-20

**Authors:** Jinkyeong Park, Dohyun Kim, Donghoon Lee, Minkyu Kim, Yoon Kim, Seon-Sook Han, Yeonjeong Heo, Hyun-Soo Choi

**Affiliations:** Department of Pulmonary, Allergy and Critical Care MedicineKyung Hee University Hospital at Gangdong, School of Medicine, Kyung Hee University 58937 Seoul 05278 Republic of Korea; ZIOVISION Company Ltd., Chuncheon Kangwon 24341 Republic of Korea; Department of Computer Science and EngineeringKangwon National University, Chuncheon 34962 Kangwon 24341 Republic of Korea; Ministry of Science and ICT Sejong 30109 Republic of Korea; Department of Internal MedicineKangwon National University, Chuncheon 34962 Kangwon 24341 Republic of Korea; Department of Computer Science and EngineeringSeoul National University of Science and Technology 65635 Seoul 01811 Republic of Korea

**Keywords:** Therapeutic drug monitoring, deep learning, vancomycin, machine learning

## Abstract

Objective: Therapeutic drug monitoring (TDM) is essential for managing medication dosages in critically ill patients, particularly for antibiotics such as vancomycin. The dynamic physiological conditions of critically ill patients require frequent monitoring of vancomycin levels to ensure therapeutic therapeutic efficacy while minimizing toxicity. Traditional Bayesian methods and pharmacokinetic (PK) models often fail because of the complex and unpredictable nature of these patients’ conditions, as well as the limitations of standard PK modeling.Methods and procedures: This study aimed to establish a gated recurrent unit (GRU)-integrated joint multilayer perceptron network (GointMLP) model to predict sequential vancomycin TDM levels in patients in the intensive care unit. The proposed model consists of three modules to maintain consistent therapeutic vancomycin concentrations while accommodating individual patient differences. By integrating regression and classification predictions, GointMLP provides a dual mechanism for clinicians to verify the reliability of predicted values for informed decision-making. Additionally, we have developed DeepTDM, a comprehensive decision support system designed for real-time vancomycin dose optimization to enhance clinical outcomes.Results: The GointMLP provides more accurate predictions compared to traditional PK models and other machine learning/deep learning approaches. This superior performance is demonstrated not only in local validation cohorts but also in the ethnically diverse MIMIC-IV dataset, validating the model’s robust generalizability.Conclusion: This work addresses the limitations of current methodologies while leveraging advancements in deep learning techniques, particularly demonstrating the effectiveness of GointMLP in enhancing patient outcomes through precise TDM. Efforts are underway to integrate DeepTDM into clinical practice, with the anticipation that it will not only support clinicians in decision-making but also substantially improve therapeutic outcomes for patients undergoing vancomycin therapy. Clinical and Translational Impact Statement: The proposed model and software enable individualized vancomycin dosing for critically ill patients, improving precision dosing and supporting seamless integration into clinical workflows

## I. Introduction

In critically ill patients receiving vancomycin, therapeutic drug monitoring (TDM) plays a crucial role in ensuring both treatment efficacy and patient safety. The complex physiological status of these patients necessitates frequent monitoring of vancomycin concentrations at multiple time points: prior to drug administration for baseline assessment, post-administration for peak and trough concentration evaluation, throughout the treatment course for ongoing monitoring, and following therapy completion to evaluate drug clearance patterns [1]. This comprehensive monitoring approach is particularly critical due to the dynamic nature of critically ill patients, who experience significant fluctuations in their physiological parameters, including variations in organ function, fluid status, and protein binding, all of which can substantially impact drug pharmacokinetics [2].

Traditional pharmacokinetic (PK) modeling approaches have demonstrated significant limitations in accurately predicting drug concentrations in critically ill populations. These models typically rely on population-based parameters derived from stable patients, failing to account for the marked physiological perturbations characteristic of critical illness [3]. While Bayesian estimation methods have emerged as an alternative approach for individualized dosing strategies, their implementation faces several practical challenges in intensive care settings. These challenges include the need for extensive and precise data collection, substantial computational resources, and specialized expertise in pharmacometric analysis. Furthermore, the rapid fluctuations in patient condition often observed in intensive care units (ICUs) make it difficult to maintain the steady-state assumptions underlying many Bayesian approaches [4], [5].

The emergence of artificial intelligence (AI) technologies, particularly in the domains of machine learning (ML) and deep learning (DL), has opened new avenues for improving TDM practices. These advanced computational approaches offer several advantages over traditional statistical methods, including their ability to efficiently process and integrate diverse patient-specific data streams [6], [7], [8], [9]. Such data may encompass demographic characteristics, temporal biochemical markers, hemodynamic parameters, and detailed treatment histories, enabling more accurate predictions of vancomycin concentrations. However, conventional ML algorithms, particularly those based on decision trees or random forests, often struggle with the temporal nature of clinical data, requiring frequent model retraining as new observations become available. Deep learning architectures, especially recurrent neural networks (RNNs), have demonstrated superior capability in processing sequential data, making them particularly well-suited for modeling time-dependent drug concentration patterns [10].

In response to these challenges, we propose a novel deep learning architecture: the Gated Recurrent Unit-integrated Joint Multilayer Perceptron Network (GointMLP) for vancomycin TDM in critically ill patients. Our model’s architecture comprises three fundamental components: (1) a sequential processing module utilizing stacked GRUs to capture temporal dependencies in patient data, (2) a joint feature extraction module employing multiple interconnected multilayer perceptron (MLP) networks to identify critical patterns in drug concentration dynamics, and (3) an advanced decision module that simultaneously performs regression and ordinal classification tasks to enhance the model’s predictive accuracy and reliability.

To facilitate clinical implementation, we have developed DeepTDM, a comprehensive decision support system designed for real-time vancomycin dose optimization. This system seamlessly integrates with existing hospital electronic medical record (EMR) infrastructure to automatically extract relevant patient data, generate accurate predictions of vancomycin trough concentrations, and provide evidence-based dosing recommendations. To rigorously evaluate its clinical impact, we are currently conducting a single-center, single-arm, retrospective confirmatory clinical trial comparing DeepTDM’s performance with conventional PK-based dosing methodologies in ICU patients.

This research seeks to establish a new paradigm in antimicrobial therapy by demonstrating the practical advantages of deep learning approaches in TDM. Through comprehensive model validation and clinical evaluation, we aim to provide a robust, AI-driven solution for optimizing vancomycin therapy in critically ill patients, ultimately contributing to improved patient outcomes in intensive care settings.

## II. Methodology

### A. Study Population

This retrospective study reviewed the medical records of 4,380 critically ill patients admitted to medical ICUs from January 1, 2010, to February 28, 2022, at a Dongguk University Ilsan Hospital (DUIH) and a Kangwon National University Hospital (KNUH) and from 2008 to 2019, at a Medical Information Mart for Intensive Care (MIMIC)-IV dataset [11]. This study was approved by the institutional review boards of DUIH (DUIH 2021-08-015) and KNUH (KNUH-2022-03-020). Internal and external validation data were obtained from the DUIH, KNUH, and MIMIC-IV datasets. The internal validation dataset included 727 patients, and the external validation dataset included 3,653 patients. We focused on patients aged over 18 years who received IV vancomycin and underwent at least one vancomycin TDM test. We primarily considered the first TDM trough level for each patient. However, for those with normal renal function and a gap of 2 weeks or more between vancomycin treatments, subsequent TDM values were treated as independent and included in the analysis. Patients treated with oral vancomycin or those under 18 years were excluded from the study. A fluorescence immunoassay was employed to measure serum vancomycin concentrations (VANC3, Cobas c 702; Roche Diagnostics, IN, USA).

The total vancomycin dose administered to all patients was 39,459. Vancomycin TDM was performed using 9,219 measured values used for analysis. Baseline characteristics showed significant differences between intensive care units in the Korean and MIMIC-IV datasets (Table 1). Younger and heavier patients were included in the MIMIC-IV dataset. In addition, this dataset displays higher TDM values. The differences between the Korean datasets were insignificant; however, the external validation dataset included a higher proportion of elderly patients.

**TABLE 1:** Baseline Characteristics of all Enrolled Patients

	Overall	Internal dataset	External dataset From KNUH	External dataset From MIMIC-IV	*P*-value^1^
Number of patients	4,380	727 (16.6)	1,211 (27.6)	2,442 (55.8)	
Male, n (%)	2,597 (59.3)	458 (63.0)	737 (60.9)	1,402 (57.4)	0.011
Age (year), (median [IQR])	70.00 [58.00, 79.00]	72.00 [59.00, 80.00]	75.00 [62.00, 81.00]	67.00 [56.00, 77.75]	<0.001
Body Weight (kg), (median [IQR])	68.00 [55.00, 83.50]	54.80 [47.40, 64.25]	58.00 [49.79, 67.00]	79.50 [66.40, 96.00]	<0.001
Race, n (%)					<0.001
ASIAN	1,991 (45.5)	727 (100.0)	1,211 (100.0)	53 (2.2)	
BLACK	227 (5.2)	0 (0.0)	0 (0.0)	227 (9.3)	
HISPANIC	83 (1.9)	0 (0.0)	0 (0.0)	83 (1.9)	
OTHER	92 (2.1)	0 (0.0)	0 (0.0)	92 (2.1)	
UNKNOWN	369 (8.4)	0 (0.0)	0 (0.0)	369 (8.4)	
WHITE	1,618 (36.9)	0 (0.0)	0 (0.0)	1,618 (36.9)	
Dialysis, n (%)	238 (5.41)	37 (5.09)	16 (1.32)	185 (7.58)	
Loading of vancomycin, n (%)	135 (0.3)	135 (1.1)	0 (0)	0 (0)	
Max accumulated dose (median [IQR])	3,750 [2,000, 6,000]	5,100 [3,000, 9,000]	5,000 [3,000, 8,000]	3,000 [2,000, 4,500]	<0.001
Max frequency (median [IQR])	4.00 [3.00, 10.00]	9.00 [5.00, 19.00]	8.00 [4.00, 16.00]	3.00 [2.00, 6.00]	<0.001
Mean dose per TDM (median [IQR])	900.00 [533.33, 1000.00]	585.71 [400.00, 1000.00]	687.50 [428.57, 1000.00]	1,000.00 [725.57, 1,000.00]	<0.001
N-th TDM, n (%)					
1	4,754 (51.0)	953 (48.7)	1,379 (48.0)	2,422 (53.9)	
2	2,246 (24.1)	495 (25.3)	716 (24.9)	1,035 (23.0)	
3	1,175 (12.6)	279 (14.3)	424 (14.8)	472 (10.5)	
4	636 (6.8)	153 (7.8)	234 (8.1)	249 (5.5)	
$\geq 5$	509 (5.4)	76 (3.9)	120 (4.2)	313 (7.0)	
Frequency of TDM per patient (median [IQR])	1.00 [1.00, 3.00]	2.00 [1.00, 3.00]	2.00 [1.00, 3.00]	1.00 [1.00, 2.00]	<0.001
Time between vancomycin injection and TDM (hours), (median [IQR])	12.00 [10.70, 22.25]	12.00 [11.50, 22.52]	11.80 [11.45, 14.88]	13.08 [9.72, 22.58]	<0.001
Serum trough value (mg/L), (median [IQR])	16.16 [11.00, 21.60]	14.00 [9.80, 19.60]	14.76 [9.10, 20.82]	17.90 [13.30, 22.60]	<0.001
Vancomycin levels, n (%)					
Sub-therapeutic	4,070 (43.7)	1,092 (55.8)	1,476 (51.4)	1,502 (33.4)	
Therapeutic	2,340 (25.1)	406 (20.8)	597 (20.8)	1,337 (29.8)	
Toxin	2,910 (31.2)	458 (23.4)	800 (27.8)	1,652 (36.8)	
Serum creatinine ( $\mu $mol/L), (median [IQR])	0.90 [0.60, 1.50]	0.69 [0.48, 1.09]	0.70 [0.50, 1.00]	1.20 [0.80, 2.00]	<0.001
Volume distribution (L), (median [IQR])	45.99 [37.80, 56.84]	38.50 [33.04, 44.82]	40.04 [34.30, 46.20]	56.00 [47.18, 67.76]	<0.001
Adjusted volume distribution (L), (median [IQR])	41.15 [38.48, 44.62]	38.96 [36.95, 41.11]	39.76 [37.93, 41.91]	43.99 [40.78, 47.58]	<0.001
CrCl (minutes), (median [IQR])	68.89 [40.81, 108.44]	73.47 [43.31, 113.09]	74.18 [45.92, 111.11]	63.16 [37.88, 104.24]	<0.001
The elimination rate constant at infusion time (Kt), (median [IQR])	0.88 [0.62, 1.25]	0.88 [0.62, 1.24]	0.86 [0.60, 1.22]	0.89 [0.62, 1.29]	<0.001
eGFR MDRD (median [IQR])	77.77 [40.16, 127.63]	103.99 [60.04, 160.97]	108.51 [64.35, 156.90]	53.91 [30.19, 90.45]	<0.001
eGFR CKDEPI (median [IQR])	80.31 [40.95, 103.31]	93.31 [61.63, 111.64]	92.75 [65.78, 108.26]	56.06 [30.55, 90.39]	<0.001
Vancomycin clearance (median [IQR])	1.52 [0.89, 2.91]	0.98 [0.61, 1.45]	0.97 [0.63, 1.37]	2.83 [1.79, 4.53]	<0.001

CKD-EPI = Chronic Kidney Disease Epidemiology Collaboration, CrCl= creatinine clearance, eGFR = estimated glomerular filtration rate, MDRD= Modification of Diet in Renal Disease, MIMIC-IV = Medical Information Mart for Intensive Care IV, TDM = therapeutic drug monitoring, CL = Vancomycin clearance, VD = Volume distribution, *P*-value^1^= comparison of the internal dataset with the external dataset from MIMIC-IV.

In contrast to the open MIMIC-IV dataset, medical datasets incur substantial costs in collection and processing, making the model’s generalization performance crucial. In this study, we evaluated our model’s generalization capability using the Korean dataset (KNUH) with a similar population to the training data and the MIMIC-IV dataset, which encompasses diverse ethnic populations with significant characteristic differences. This evaluation suggests the robust generalization capability of the proposed model, ensuring its applicability across various clinical settings and patient demographics.

### B. Data Processing

In our experiment, the variables used were based on those from previous studies [12] but were modified and added based on clinician advice to predict sequential TDM levels. Sixteen variables were selected to predict vancomycin trough concentrations. These variables included sex, age, height, body weight, the interval between each dose of vancomycin, time from vancomycin injection to blood collection for TDM, use of a loading dose, dialysis status, serum creatinine levels(scr), average volume of distribution, elimination rate constant, vancomycin administration time, the dose of vancomycin per infusion, and cumulative dose per TDM. Additionally, given that patients with impaired renal function may have vancomycin metabolism and elimination issues, eGFR was used as a variable. We employed the modification of diet in renal disease (MDRD) formula to calculate GFR, which reflects renal function [13]. We calculated creatinine clearance (CrCl) using the equation proposed by Cockcroft and Gault [14] to estimate vancomycin clearance, as shown below:

\begin{equation*} CrCl_{\text {mL/min}} = \frac {(140 - \text {Age}) \times \text {Weight (kg)} \times K}{(72 \times \text {Scr (mg/dL)})} \tag {1}\end{equation*}

where *K* is a sex-specific correction factor, assigned a value of 1.0 for males and 0.85 for females.

Vancomycin clearance was derived from CrCl using the linear relationship $CL_{\text {vanco}} = a \times CrCl + b$. Here, *a* is the slope, reflecting the proportional contribution of renal function to vancomycin clearance, and *b* is the intercept, accounting for minimal non-renal clearance (e.g., hepatic metabolism). Based on established pharmacokinetic literature [15], we calculated vancomycin clearance as below. These coefficients were not derived from vancomycin concentration data but from prior population pharmacokinetic studies, ensuring independence from the target variable.

\begin{align*} CL_{\text {vanco}} & = \left ({{0.695 \times \frac {CrCl_{\min }}{\text {Weight (kg)}} + 0.05 }}\right) \\ & \quad \times \text {Weight (kg)} \times 0.06 \tag {2}\end{align*}

Patients lacking essential data for PK/PD calculations (e.g., height, weight, creatinine), those with incomplete parameters, or without clearly defined vancomycin trough levels were excluded from the study. Furthermore, missing values were imputed using forward filling, in which the most recent available measurements before each vancomycin administration time were carried forward. If missing values remained after this procedure, the corresponding records were excluded from the analysis. These criterion was consistently applied to both the Korean and the MIMIC-IV datasets. As a result, all datasets consisted of complete data without missing values. The PK parameters were recalculated at each vancomycin dosing time point using the most recent clinical data available in the EMR. In particular, vancomycin levels were updated at time points where actual trough concentrations were recorded. By ensuring that all variables, including the PK parameters used in this study, were derived from the most up-to-date values, we sought to maintain fairness in model comparisons.

In the data preprocessing, we normalized all continuous variables to a range of [−1, 1] using the Min-Max scaler. In contrast, the two binary categorical variables (vancomycin loading and dialysis status) remained in their original form to avoid increasing additional costs. To perform ordinal regression for vancomycin concentration levels, we categorized vancomycin trough concentrations into three groups based on their ranges: sub-therapeutic range (0- $15~\mu $g/mL), therapeutic range (15- $20~\mu $g/mL), and toxic range ( $> 20~\mu $g/mL). To create ordinal categories, we marked each applicable category with one and transformed it into a sequence of binary labels. Finally, to enhance learning efficiency, we limited the maximum sequence length to 25 during the training phase, whereas no restriction was applied during the evaluation phase.

### C. Proposed Model, Gointmlp

**FIGURE 1.**
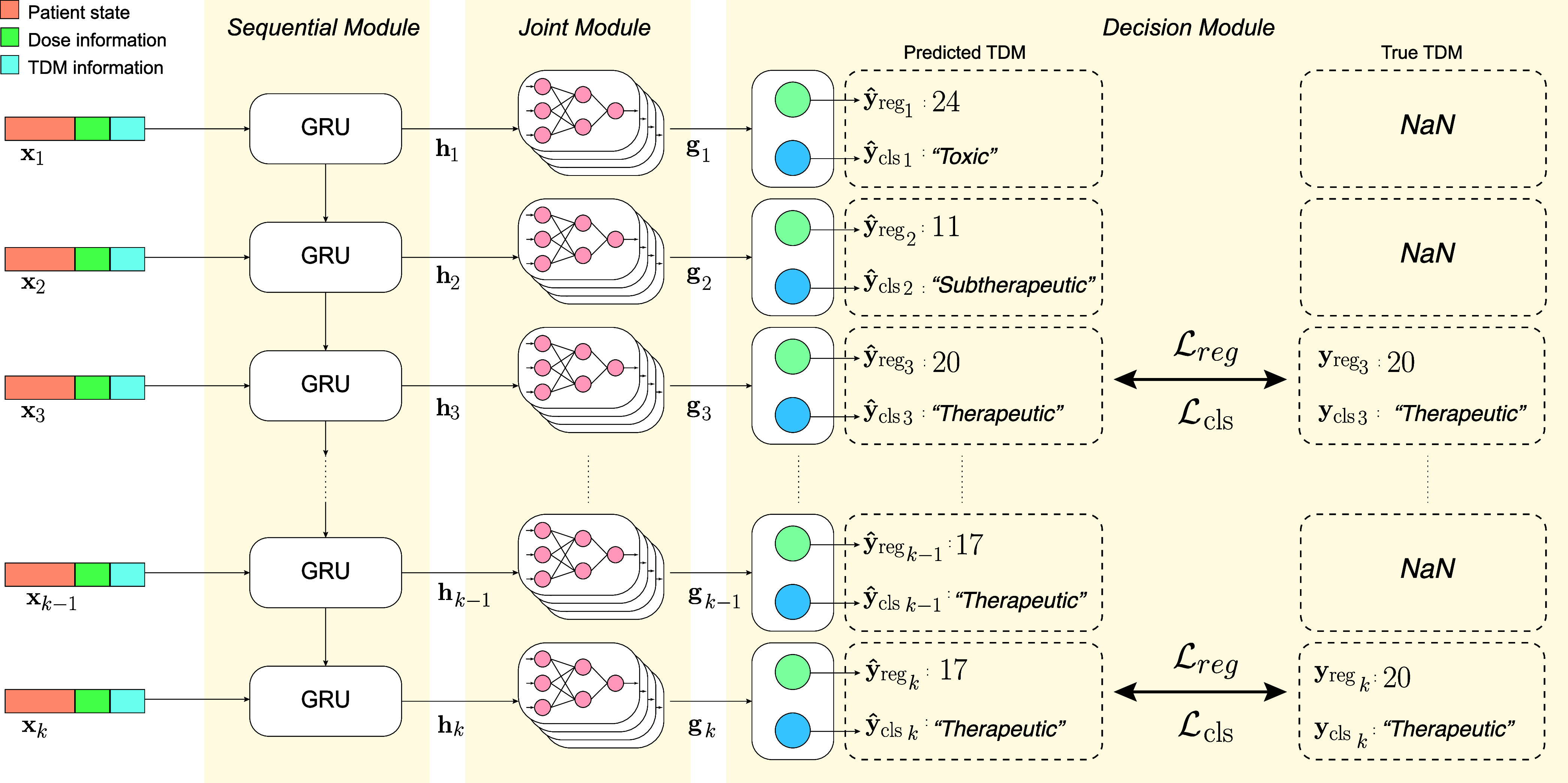
The architecture of GRU-integrated joint multilayer perceptron network (GointMLP). The GointMLP model consists of three modules. The sequential and joint modules efficiently extract features of variables and temporal information. The decision module predicts vancomycin trough concentrations and levels. Also, for efficient learning, the model’s weights are updated only at timesteps where actual vancomycin trough concentrations exist.

We propose GointMLP as a predictor of sequential vancomycin TDM levels in patients in the intensive care unit. The GoinMLP model predicts vancomycin trough levels at all dosing time points (Fig. 1), enabling evaluation of the predicted values at each administration. However, model weight updates were performed only at time points where actual trough level measurements were available. The GointMLP model has three key components: 1) a sequence module designed to capture temporal information, 2) a joint module focused on effectively representing drug concentration features, and 3) a decision module aimed at regressing the vancomycin trough concentration and predicting drug concentration levels, thereby helping clinicians with their decision-making processes. By integrating the advantages of each module, the proposed model demonstrated superior performance compared to other models. Fig. 1 shows the architecture of the proposed model.

#### 1) Sequential Module

Since most of the tabular medical data is sequential, handling temporal information is essential. However, most traditional methods are inappropriate because they are not designed to handle time-series data. Various models have been introduced with the advancement of deep-learning technologies. Notably, RNN demonstrated remarkable performance with sequential data. In particular, despite being a less complex model within the RNN family, the GRU is known for its fast training speed and good performance, even with a small dataset [16]. Consequently, the proposed model incorporated the stacked GRU architecture as the sequence module. The mathematical formulation of the stacked GRU model is as follows:

\begin{align*} z_{t}^{i} & = \sigma (W_{z}^{i} \cdot [h_{t-1}^{i},h_{t}^{i-1} ]) \\ r_{t}^{i} & = \sigma (W_{r}^{i} \cdot [h_{t-1}^{i},h_{t}^{i-1} ]) \\ \tilde {h}_{t}^{i} & = \tanh (W^{i} \cdot [r_{t}^{i} \odot h_{t-1}^{i},h_{t}^{i-1}]) \\ h_{t}^{i} & = (1-z_{t}^{i}) \odot h_{t}^{i-1} + z_{t}^{i} \odot h_{t-1}^{i} \tag {3}\end{align*}

where $\sigma $ denotes the sigmoid activation function. The operations $\cdot $ and $\odot $ represent matrix multiplication and element-wise matrix multiplication, respectively. $z_{t}^{i}$ and $r_{t}^{i}$ are the update and reset gates of the ith GRU at time step *t*, respectively. Furthermore, $W_{z}^{i}$ and $W_{r}^{i}$ denote the weights of the update and reset gates of the ith GRU, respectively. The term $h_{t}^{i}$ denotes the output of the hidden layer. Note that $h_{t}^{0}$ in the first layer corresponds to the input variables. Finally, $\tilde {h}_{t}^{i}$ denotes the output of the candidate hidden layer for the ith GRU at time step *t*, which encodes the historical information from the hidden layers.

#### 2) Joint Module

A joint multilayer Perceptron Network (JointMLP) is a model that combines multiple MLP networks, comprising shallow networks that have a lower risk of overfitting and exhibit good model accuracy and robustness [12]. Each MLP in the architecture consists of multiple hidden layers and utilizes a LeakyReLU activation function. The formula for MLP is as follows:

\begin{equation*} g_{l}^{i}=\mathrm {LeakyReLU}(W_{l}^{i} \cdot x_{l-1}^{i} + b_{l}^{i}) \tag {4}\end{equation*}

where $W_{l}^{i}$ refers to the weight matrix of the lth layer in the ith MLP network, and $x_{l-1}^{i}$ and $b_{l}^{i}$ denote the input and bias, respectively. Specifically, within each network, an attention layer was utilized as the first multilayer perceptron layer to focus on the key features of the inputs. The inputs to the JointMLP model were the output features from the last layer of the GRU for each time step *t*. The formula used is as follows:

\begin{equation*} g'{}_{1,t}^{i}=\mathrm {Sparsemax}(g_{1,t}^{i} \odot p^{i}) \tag {5}\end{equation*}

Within the *i*-th MLP network, $g_{1,t}^{i}$ denotes the outputs of the hidden layer from the first layer, and $p^{i}$ represents the prior vector used for adjusting the weights. Sparsemax is an alternative to the softmax function designed to make the distribution of possible outcomes sparse. This characteristic improves the performance of the attention mechanism.

#### 3) Decision Module

To predict the vancomycin trough concentration and levels, the outputs of each independent MLP were concatenated and then fed into a decision module. The decision module comprises a regression layer for predicting vancomycin trough concentrations and an ordinal regression layer for classifying concentration ranges. By incorporating a decision module, the proposed model learns two tasks simultaneously: regression and classification. This multi-task learning approach introduces an inductive bias, as the model learns to extract shared essential features for both tasks, potentially leading to better generalization ability. We constructed a weighted objective function by combining the losses from each task layer. The objective function is formulated as follows:

\begin{align*} L= M \odot L_{reg}(F_{r}(G_{t}),Y_{reg_{t}}) + \lambda (M \odot L_{cls}(F_{c}(G_{t}),Y_{cls_{t}})) \tag {6}\end{align*}

where $M\in [{0,1}]$ denotes a mask indicating whether TDM values are available at each time step, based on actual blood samples. The mask helps prevent the model from unnecessarily updating its weights when TDM data are missing, ensuring that it learns only from valid data points. $F_{r}$ and $F_{c}$ represent the regression and ordinal regression layers, respectively. $L_{reg}(\cdot,\cdot)$ denotes the regression objective function, defined as the mean square error. $G_{t}$ is formed by concatenating the outputs of multiple MLPs at time step *t*. $L_{cls}(\cdot,\cdot)$ denotes the cross-entropy loss or ordinal regression loss for classification, where $\lambda $ is a balancing weight between regression and classification losses. In this study, we used ordinal regression loss as the cross-entropy of K-1 binary classifiers:

\begin{equation*} L_{cls}=-\Sigma _{k=1}^{k-1}y_{k} log \hat {y}_{k} + (1-y_{k})log(1-\hat {y}_{k}) \tag {7}\end{equation*}

where $\hat {y}_{k}$ denotes the sigmoid function output of the ordinal regression layer, and K is the number of classes.

### D. Evaluation Methods

The primary endpoint was predictive performance of vancomycin levels within the therapeutic range. The internal and external datasets’ baseline variables and patient characteristics are presented as frequencies with percentages or mean values with standard deviations. Between-dataset comparisons were performed using the paired t-test for continuous variables and the chi-square test for categorical variables. The measured serum vancomycin levels were used as the true values. To evaluate vancomycin trough concentration predictions, we utilized four model types: the population pharmacokinetic (PPK) model from the infectious disease management program website at UCS [17], machine learning model (XGBoost), and deep learning models (TabNet, JointMLP, GRU, and GointMLP). Each model’s bias and precision were assessed using root mean squared error (RMSE) and coefficient of determination ( $R^{2}$). RMSE is a widely utilized metric that assesses the average magnitude of differences between predicted values and actual observations. Equation (6) defines the RMSE, in which $\hat {y}_{\text {reg}}^{i}$ and $y_{\text {reg}}^{i}$ denote the predicted vancomycin trough level and the actual measurement for the *i*-th observation, respectively.

\begin{equation*} RMSE=\sqrt {\frac {\Sigma _{i=1}^{n}(\hat {y}_{\text {reg}}^{i} - y_{\text {reg}}^{i})^{2}}{n}} \tag {8}\end{equation*}

To complement RMSE, which only quantifies absolute prediction errors, we employed the $R^{2}$ as an additional evaluation metric to assess each model’s capability to explain the variability in the data. The $R^{2}$ is formulated as follows:

\begin{equation*} R^{2} = 1 - \frac {\sum _{i=1}^{n} (y_{\text {reg}}^{i} - \hat {y}_{\text {reg}}^{i})^{2}}{\sum _{i=1}^{n} (y_{\text {reg}}^{i} - \bar {y}_{\text {reg}})^{2}} \tag {9}\end{equation*}

where $\hat {y}_{\text {reg}}^{i}$ and ${y}_{\text {reg}}^{i}$ represent the predicted value and the actual value for the *i*-th observation, respectively, and $\bar {y}_{\text {reg}}$ denotes the mean of actual values. To evaluate the statistical significance between the models, paired t-tests were employed on the mean absolute error (MAE) to assess differences in prediction performance. P-values of <.05 were considered statistically.

### E. Model Implementation

We implemented and evaluated various models using R v.3.4.4, Python v3.10.13, SciPy v.1.11.4, PyTorch v.2.1.0, and PyTorch Lightning v.1.9.0 libraries. For the XGBoost implementation, we employed XGBoost v1.7.3 library with the following configuration: learning rate (eta) of 0.2, minimum loss reduction (gamma) of 0.001, and other hyperparameters were set to default values. For TabNet implementation, we utilized the pytorch-tabnet v4.1.0 library with the following parameters: maximum training epochs of 1000 with a virtual batch size of 64. Early stopping was applied with a patience of 500 epochs, and RMSE was used as the evaluation metric.

The JointMLP architecture followed the implementation described in [12], configured with maximum epochs of 1000, training batch size of 512, and learning rate of 1e-4. Other hyperparameters were set to follow [12]. We used a 2-layer stacked GRU with 12 hidden units and a maximum sequence length of 20 for the GRU implementation. The model was trained for 1000 epochs with a batch size 512 and a learning rate 1e-4.

The baseline models were set heuristically based on commonly used parameter settings. However, our proposed model has a more complex structure, making manual parameter setting difficult. To address this, we employed optuna’s bayesian optimization with conjunction k-fold cross-validation to optimize the model’s hyperparameters. The hyperparameter ranges were defined as follows: learning rate from 1e-5 to 1e-2, maximum sequence length of GRU from 1 to 50, the number of hidden units in each GRU from 10 to 20, number of stacked GRU layers from 3 to 6, number of MLPs in JointMLP from 10 to 20, number of hidden layers in each MLP from 5 to 10, and hidden units in the hidden layers from 32 to 128. Subsequently, the sequential module and joint module of GointMLP were implemented as follows: (1) a sequential module implemented as a 3-layer stacked GRU with 15 hidden units and a maximum sequence length of 20, and (2) a joint module comprising 15 different MLPs, where each MLP consists of 5 hidden layers with 64 hidden units. The model was trained for 1000 epochs with a batch size of 512 and a learning rate of 5e-4. The balancing weight ( $\lambda $) of the GointMLP with the decision module was set to 0.29 through manual parameter tuning.

To prevent overfitting of JointMLP, GRU, and GointMLP models, early stopping was applied with patience of 50 epochs and warm-up steps of 300 epochs. Gradient clipping was applied only to the GRU and GointMLP with the decision module, as it degraded the performance of the other deep learning models like JointMLP and GointMLP without the decision module. The maximum norm of the gradients was set to 0.5 heuristically for gradient clipping.

## III. Results

A deep learning model was developed for vancomycin TDM using data from 727 critically ill patients enrolled in DUIH. This dataset, before pre-processing, was divided into a training set of 545 patients across 764 cases and a test set comprising 182 patients in 189 cases, which represents approximately 25% of the total internal dataset. For external validation, we used 1,379 cases from 1,211 patients at KNUH and 2,557 cases from 2,442 patients in the MIMIC-IV dataset. Additionally, We used 5-fold cross-validation on the internal training dataset to assess the generalization capabilities of the model. All training and tests were conducted on the windows 11 operating system with Windows Subsystem for Linux (WSL), using a system equipped with an Intel(R) Core(TM) i5-12600 CPU processor with a base frequency of 3.60 GHz, 62 GB ( $2\times 32$ GB) DDR4-3200 MHz RAM, and an NVIDIA GeForce RTX 3060 GPU with 12 GB GDDR6 memory.

### A. Comparative Models

We compared four other models and two modules within the GointMLP network: the PPK model, extreme gradient boosting (XGBoost), TabNet, sequential module, and joint module. 1) The PPK is a pharmacokinetic model that evaluates the correlation between the concentration of a drug in the body and time [18]. 2) Linear regression is a simple and widely used method for analyzing tabular data. It models the linear relationship between dependent and independent variables. 3) XGBoost is a suitable model for learning from tabular data. This model is required for distributed training and regularization [19]. 4) TabNet, a state-of-the-art deep learning model for analyzing tabular datasets, prioritizes important features using attention mechanisms sequentially [20]. 5) The GRU, used as the sequential module in this experiment, is a type of neural network architecture specialized for processing time data, offering advantages in capturing temporal dependencies. In this study, it was crucial to handle temporal information effectively to predict vancomycin trough concentrations sequentially, making the GRU a key component. 6) For the joint module, we utilized the JointMLP, which demonstrated the best performance in predicting vancomycin trough concentrations in the initial TDM compared to the other models.

Each fold of the experimental models was evaluated using both internal and external validation datasets. The metrics obtained from these tests were used to calculate the 95% confidence intervals across the five folds, as listed in Table 2. As shown in Table 2, the proposed GointMLP model demonstrated superior performance compared to traditional PPK model and other machine learning and deep learning approaches on both internal and external validation datasets. For the internal validation dataset, GointMLP achieved an RMSE of 6.70. These results indicate that the proposed model not only minimizes prediction errors but also effectively explains the variance in the data among all compared models. The external validation datasets demonstrated an RMSE of 8.99 on the domestic Korean dataset and an RMSE of 9.02 on the MIMIC-IV dataset, respectively. These findings suggest the robust generalization capability of our proposed model, as it achieved superior performance not only on the domestic Korean dataset but also on the MIMIC-IV dataset, which encompasses diverse ethnic populations.

**TABLE 2:** Comparative Results Via Cross-Validation

Model		RMSE (95% CI)				R2 (95% CI)			
		Internal	External	MIMIC-IV	MIMIC-IV*	Internal	External	MIMIC-IV	MIMIC-IV*
PPK		13.33 (8.86, 17.83)	13.80 (12.31, 15.89)	31.85 (17.81, 53.30)	-	-1.85 (-4.79 -0.32)	-0.70 (-1.33 -0.31)	-15.79 (-48.01 -4.29)	-
Linear		7.62 (7.55, 7.69)	9.82 (9.75, 9.88)	9.68 (9.60, 9.75)	8.10 (8.07, 8.12)	0.07 (0.05, 0.09)	0.14 (0.13, 0.15)	-0.35 (-0.38, -0.33)	0.05 (0.05, 0.06)
XGBoost		7.62 (7.49, 7.75)	9.03 (8.98, 9.08)	8.82 (8.52, 9.13)	8.15 (8.08, 8.22)	0.07 (0.04, 0.10)	0.20 (0.17, 0.23)	-0.10 (-0.15, -0.05)	0.04 (0.02, 0.05)
TabNet		7.34 (7.22, 7.45)	9.77 (9.61, 9.92)	9.48 (8.91, 10.06)	8.52 (8.32, 8.71)	0.14 (0.11,0.16)	0.15 (0.12,0.18)	-0.31 (-0.47, -0.15)	-0.05 (-0.10, 0.00)
GointMLP	S	9.92 (8.58, 11.27)	12.28 (10.61, 13.96)	14.55 (10.45, 18.68)	8.89 (8.30, 9.48)	-0.62 (-1.05, -0.19)	-0.38 (-0.74, -0.01)	-2.38 (-4.22, -0.54)	-0.15 (-0.30, -0.02)
	J	7.01 (6.92, 7.11)	9.53 (9.47, 9.59)	9.88 (9.75, 10.01)	7.92 (7.88, 7.96)	0.21 (0.19, 0.23)	0.19 (0.18, 0.20)	-0.41 (-0.45,-0.38)	0.09 (0.08, 0.10)
	S+J	6.75 (6.55, 6.95)	9.14 (9.01, 9.28)	9.23 (9.02, 9.45)	7.92 (7.86,7.97)	0.27 (0.22, 0.31)	0.25 (0.23, 0.28)	-0.23 (-0.29, -0.18)	0.09 (0.08,0.11)
	S+J+D	6.70 (6.48, 6.92)	8.99 (8.89, 9.10)	9.02 (8.77, 9.26)	7.84 (7.78, 7.90)	0.28 (0.23, 0.33)	0.28 (0.26, 0.30)	-0.18 (-0.24, -0.11)	0.11 (0.10, 0.12)

RMSE: root mean squared error, MIMIC-IV*: fine-tuned (GointMLP) / trained from scratch with MIMIC-IV data, S: sequential module, J: joint module, D: decision module

### B. Ablation Results Using Gointmlp

The sequential module, a network architecture suitable for processing time-series data, showed poor performance compared to the other models in our experiment. The joint module performed well but showed a slight lack of generalization capability for external data. Despite these shortcomings, the GointMLP model, which merges sequential and joint module functionalities, surpasses the performance of other models. Furthermore, to enhance the reliability of the GointMLP model, we added a decision module that combines classification and regression layers. The experimental results confirmed that this modification led to superior performance across all datasets compared with GointMLP without this feature. This indicates that transitioning from a single regression task to a multitask approach enables the GointMLP model to extract more robust features, thereby improving its generalization performance.

### C. Fine-Tuning Results for the Mimic-Iv Dataset

Before fine-tuning, our model underperformed the XGBoost model on the MIMIC-IV dataset, with a higher RMSE score (9.02 vs. 8.82) and a negative $R^{2}$ value, indicating a less accurate representation of the dataset. This discrepancy highlights the need for model optimization to capture better the unique characteristics of the MIMIC-IV dataset, which represents an American population with distinct data distributions. In response, we strategically allocated 10% of the dataset to fine-tune our deep learning model, which was unsuitable for the XGBoost and TabNet models because of their incompatibility with fine-tuning methods. This targeted fine-tuning significantly enhanced our model’s performance, not only reducing the RMSE score to 7.84, a 306% improvement over the pre-fine-tuning performance against the PPK model (from 31.85 to 7.84) but also surpassing the performance of both the joint and sequential modules, used alone, by 1% (from 7.92 to 7.84) and 54% (from 8.89 to 7.84), respectively. The GointMLP with the decision module showed a modest performance improvement of 1% (from 7.92 to 7.84) compared to its counterpart without the decision module. Moreover, this improvement after fine-tuning is further evidenced by the shift to a positive $R^{2}$ value, affirming that our model, following fine-tuning, offers the most accurate representation of the MIMIC-IV dataset. This transformation underscores the effectiveness of fine-tuning in significantly enhancing model performance and achieving the best results on this specific dataset.

In addition, we trained models that could not be fine-tuned from scratch on a mixed dataset comprising the original training data and 10% of the MIMIC-IV dataset reserved for fine-tuning, and subsequently compared their performance. The results demonstrate that the proposed fine-tuned model consistently outperforms those trained on the mixed dataset when evaluated on the MIMIC-IV test set, thereby underscoring both the adaptability and superior effectiveness of the proposed approach.

### D. Statistical Analysis of the PPK and Gointmlp

**FIGURE 2.**
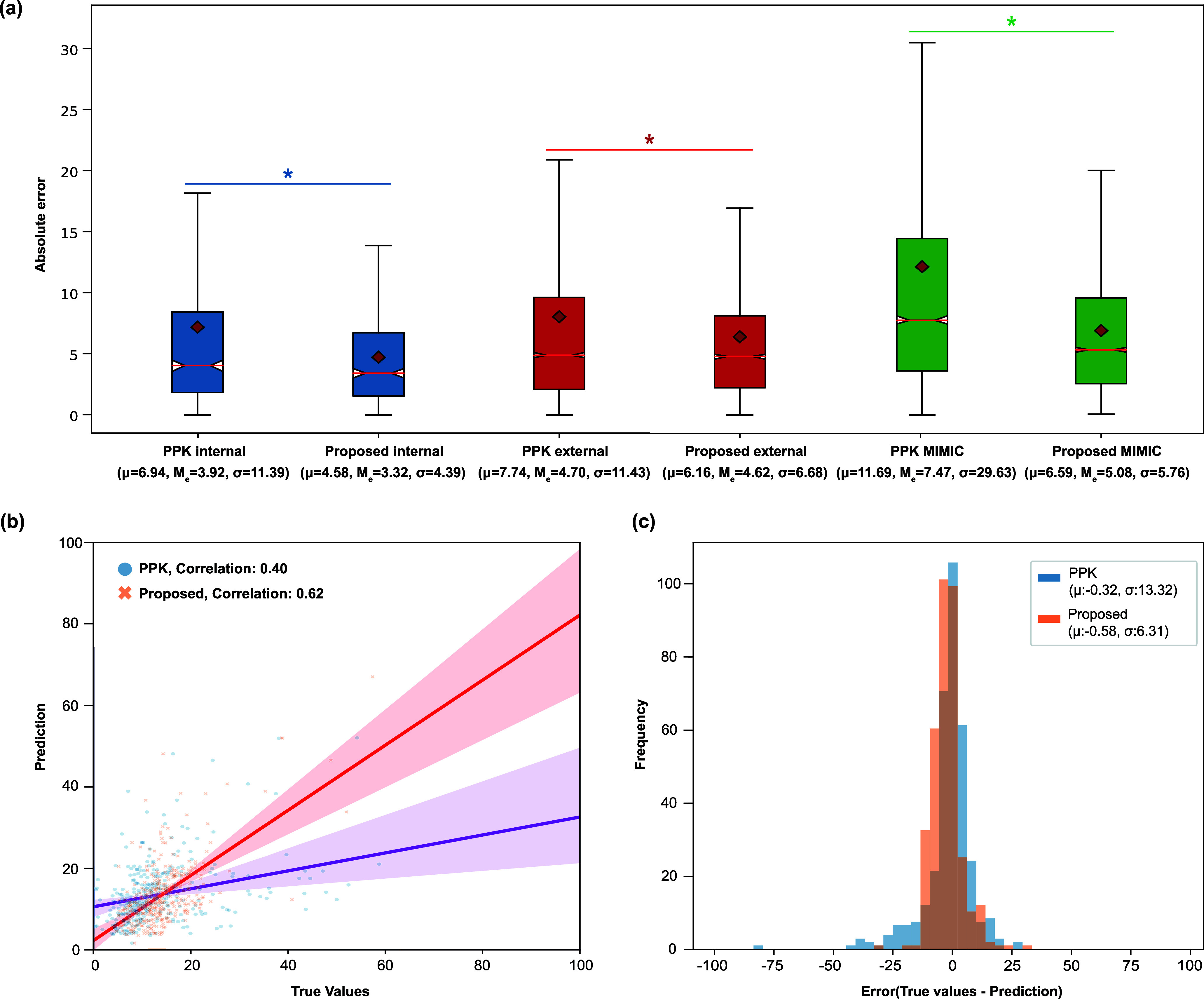
Performance comparison between PPK and the proposed model showing (a) absolute error metric Box Plots of all datasets with median (red line, $M_{e}$), mean (red rhombus, $\mu $), standard deviation ( $\sigma $) and statistical significance (asterisks, p < 0.05). (b) scatter plots comparing predicted and target values of the internal validation dataset. (c) The histogram error against the predicted and the target values on the internal validation dataset. All analyses shown in Figure 2 are based on total TDM test cases: internal (n = 383), external (n = 2,873), and MIMIC-IV (n = 4,491).

The proposed model exhibited the best predictive performance for vancomycin TDM on internal and external datasets among all the compared models. In particular, compared to PPK, it increased the predictive accuracy for post-infusion vancomycin concentration by 99% (13.33 vs. 6.70 by RMSE) in the internal dataset and 54% (13.80 vs. 8.99 by RMSE) in the external dataset. Statistical significance between the two models was confirmed (Fig. 2(a)). For all the datasets, the p-value from the paired t-test of the mean absolute error (MAE) between the two models was less than 0.05, indicating that the difference between the models was statistically significant. The proposed model demonstrated higher linear correlations (Fig. 2(b)), and the mean error and variance were closer to zero and smaller, respectively, than those of the PPK model (Fig. 2(c)).

### E. Analysis of TDM Regression and Classification Prediction

**FIGURE 3.**
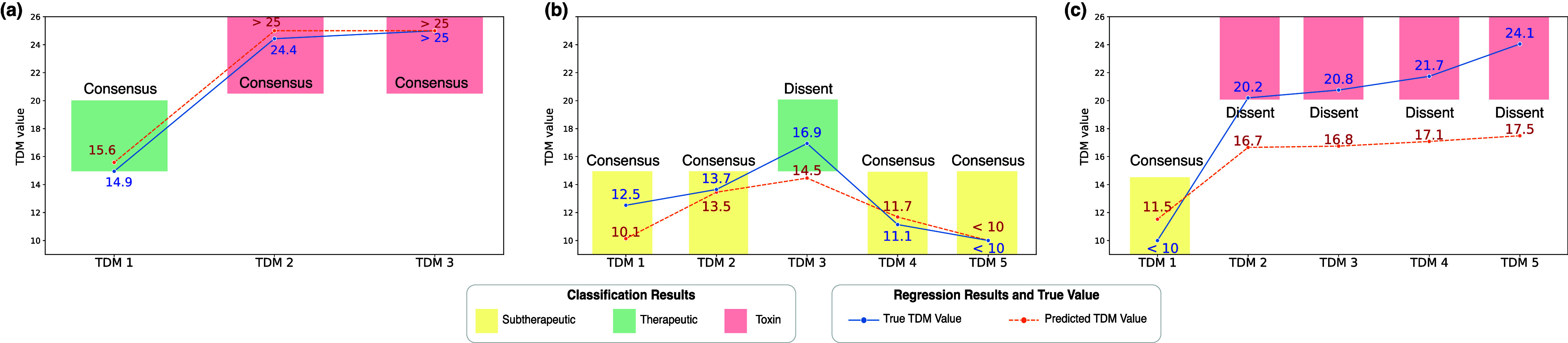
Regression plot for true and predicted values with bar plot for classifier values for each nth TDM Examples illustrate the correlation between regression predictions, classification predictions, and actual TDM values: (a) both predictions closely match actual values, (b) precise regression predictions with minor classification deviations, and (c) classification accurately reflects TDM values despite slight regression discrepancies.

To enhance output reliability, the model not only predicts vancomycin levels but also categorizes them (subtherapeutic range, therapeutic range, and toxin range) via consistent rank logs (CORAL) [21]. Fig. 3 illustrates the ordinal regressions of vancomycin and the classifier of CORAL after TDM sequencing. Alignment between the predicted concentrations and the classifier’s ordinal predictions signals high reliability, whereas discrepancies suggest lower reliability, aiding clinicians in result assessments. Fig. 3(a) illustrates that the predicted regression and classification outputs yielded identical results, indicating a consensus in the decision-making process. Fig. 3(b) demonstrates a slight discrepancy between the predicted regression and true TDM values; however, the classification was accurately predicted. This discrepancy between the regression and classification results indicates a dissent, which allows clinicians to reconsider the results. Fig. 3(c) shows that the predicted regression values of the proposed model were slightly underestimated for patients in the toxic range, leading to disagreement in the classification results. This trend demonstrates that the proposed model can serve as a reliable aid in clinical decision-making. Specifically, stable clinical decisions can be made with higher sensitivity by conservatively approaching the possibility of being in the toxin range.

### F. Feature Importance

**FIGURE 4.**
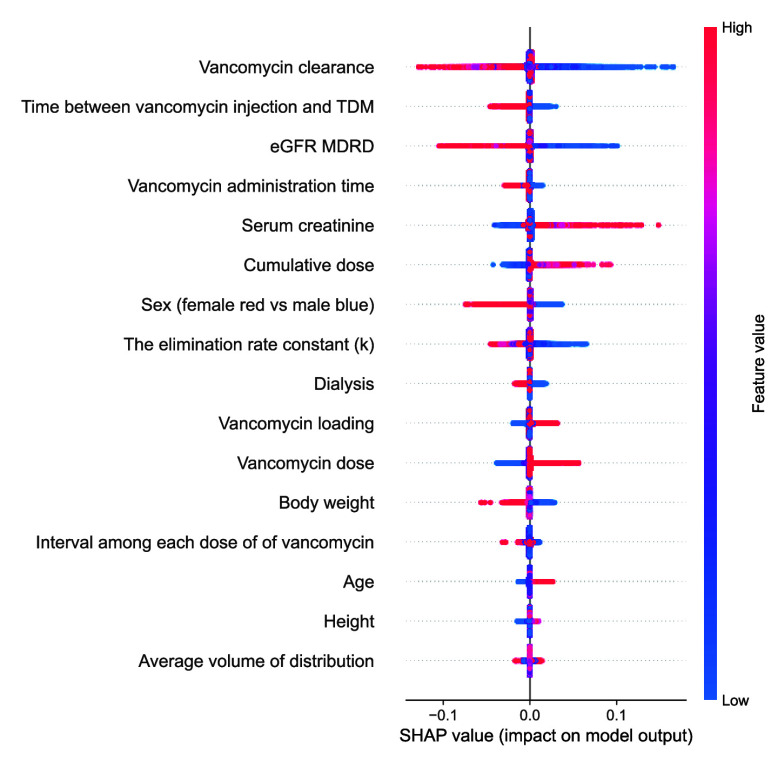
The Shapley additive exPlanations values of the proposed model on Internal test sets.

To explain the results of the proposed model, we utilized the Shapley values from Shapley Additive explanations (SHAP) to assess the feature importance [22]. In all datasets, vancomycin clearance, the time between vancomycin injection and TDM, sequential estimated glomerular filtration rate (eGFR) calculated using the MDRD formula, vancomycin administration time, serum creatinine level, and cumulative dose were consistently important for model predictions (Fig. 4). Feature importance was similar in both the internal and external datasets, with the MIMIC-IV dataset demonstrating comparable importance, albeit with slight variations.

## IV. DeepTDM

**FIGURE 5.**
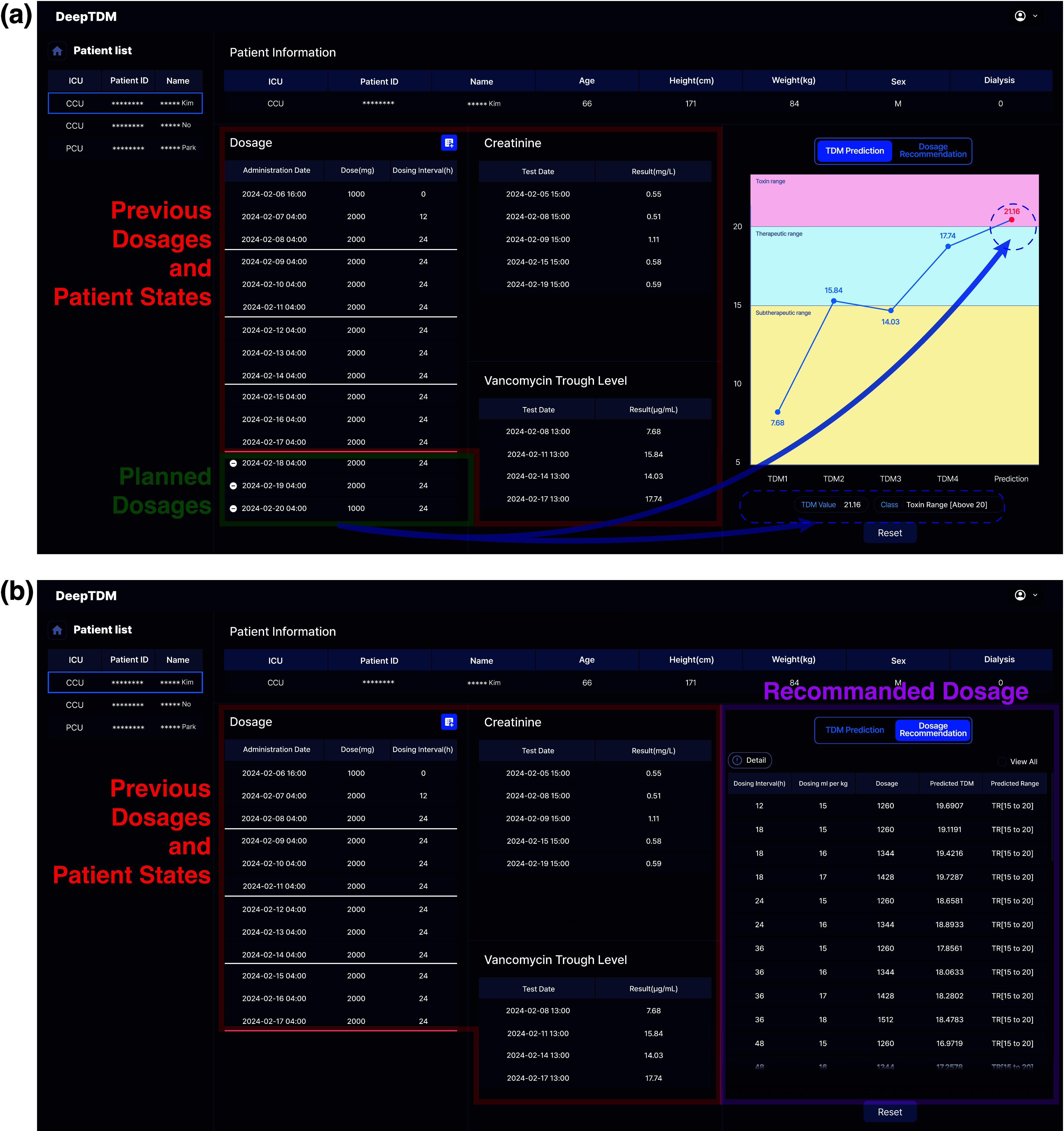
The user interface of DeepTDM. (a) TDM Prediction: the clinicians can input planned future drug doses and dosing intervals based on previous dosages and patient states, and the program then predicts the corresponding trough level based on these inputs. (b) Dosage Recommendation: DeepTDM displays only those vancomycin dose–interval combinations predicted to maintain steady-state trough levels within the therapeutic range (15–20 mg/L). By narrowing down the feasible options, the system guides clinicians toward selecting dosing regimens that are both safe and therapeutically effective.

We confirmed the significance of the proposed model’s performance with domestic and external data, including diverse racial groups. However, this study has limitations as it was conducted retrospectively and lacked evaluation of its clinical impact and usability in real-world settings. To address this, we developed a program called DeepTDM and are assessing the practical clinical applicability of the proposed GointMLP model. dependent is designed to interface with hospital electronic medical record(EMR) databases, automatically collecting demographic information, laboratory data, and medication details of intensive care unit patients. The collected data are input for the proposed GointMLP model to predict current vancomycin drug concentrations in patients. The user interface of DeepTDM is as shown in Fig. 5.

The prediction function predicts vancomycin trough levels based on critically ill patients’ information automatically collected from the EMR database. Additionally, it allows clinicians to input planned future drug doses and dosing intervals plans based on historical data, thereby simulating the patient’s anticipated trough level (Fig. 5(a)). For example, Fig. 5(a) illustrates a case with four TDM sessions and five serum creatinine measurements. Based on this historical information, the next dosing regimen can be simulated in advance, and DeepTDM enables adjustment of both vancomycin dosage and dosing intervals at each administration. Unlike conventional TDM, which generally recommends maintaining a fixed dosage and interval within a single round, DeepTDM reflects clinical practice, where regimens may change depending on patient status, thereby allowing flexible modifications at every dosing time point. In this example, vancomycin was administered three times at 24-hour intervals, and for the final administration, a different dosage of 1 mg was simulated, yielding a predicted trough concentration of 21.16 mg/L.

The dosage recommendation function was developed under the assumption that the patient had reached a steady-state trough level and required guidance for the next vancomycin dosing regimen. It simulates the patient’s expected vancomycin trough levels by combining possible future dosages and intervals based on prior TDM information. Then it filters these combinations to provide options within the therapeutic range (15–20 mg/L), thereby assisting clinicians in selecting appropriate dosing regimens. Specifically, the function suggests combinations of dosing intervals and per-kilogram doses predicted by GoinMLP to yield steady-state trough concentrations within the target range. Based on clinical expert opinion, the recommendation system differentiated between general critically ill patients and those undergoing dialysis. For general critically ill patients, dosing intervals of 8, 12, 18, 24, and 48 hours were considered, with per-kilogram doses of 15–20 in increments of 1 ml/kg. In contrast, for dialysis patients, the dosing interval was restricted to 12 hours, and reduced per-kilogram doses of 7.5–10 in 0.5 ml/kg increments were applied to account for impaired renal function. Finally, combinations of these predefined values, together with individual patient histories, were used as model inputs, and the predicted trough concentrations determined which dose–interval combinations, within a range of 15–20 mg/L were recommended to the clinician (Fig. 5(b)).

We are currently conducting a clinical trial, approved by the Korean Ministry of Food and Drug Safety, to evaluate the clinical efficacy and practical applicability of DeepTDM. This trial aims to assess the model’s performance in real-world clinical settings, validate its effectiveness compared to existing methods, and refine its usability based on clinician feedback. The primary evaluation criteria for the clinical trial are as follows: (1) Patients aged 19 years or older admitted to the intensive care unit (ICU) with a history of intravenous vancomycin treatment are included in the study. All eligible patients form a single group, and DeepTDM predictions are directly compared with conventional PK model predictions using the same EMR data. (2) The study group utilizes DeepTDM, while the control group relies on PPK for vancomycin concentration prediction. (3) Predicted trough levels are compared with actual measured values, and model performance is evaluated using MAE, RMSE, and $R^{2}$. Through this clinical trial, we aim to demonstrate the practical utility of the GointMLP model and DeepTDM program in real-world clinical environments, paving the way for a new paradigm in personalized drug administration optimization. Additionally, this study is expected to serve as a foundational case for integrating artificial intelligence-based decision support systems into clinical practice for critically ill patients.

## V. Discussion

For a consistent decision-making system, this study suggests using DeepTDM with GointMLP to ensure it is not influenced by clinicians’ abilities. The model was applied to predict the appropriate vancomycin trough concentration in critically ill patients requiring vancomycin treatment under various situations.

In this study, we compared the performances of several learning models in predicting sequential vancomycin levels. XGBoost and TabNet are well suited for handling tabular data, whereas the GRU of the sequence module is widely used for efficiently processing sequential data. Specifically, JointMLP, utilized as the joint module, has been proven to excel in predicting initial vancomycin trough concentrations, as demonstrated in our previous research. However, as models other than GRU are not well suited for handling temporal information, our proposed model, GointMLP, aims to appropriately handle temporal information to consistently maintain TDM-level prediction performance. The GointMLP model was designed to combine the sequence and joint modules, leveraging the sequence module’s ability to process sequential data and the joint module’s effectiveness in handling pervasive noisy medical data. Furthermore, extending from a regression problem to adding classification tasks, the proposed model effectively handled multitask problems and demonstrated improved accuracy compared with the regression problem alone.

Compared with previous studies, GointMLP performed better in accurately predicting vancomycin levels. Specifically, our model outperformed all the other models on the Korean critically ill patient dataset but did not perform best on the MIMIC-IV dataset. Nonetheless, the negative $R^{2}$ values across all the experimental models indicate the difficulty in generalizing the performance to the MIMIC-IV dataset, which has a significantly different distribution from the internal dataset. To address this issue, we used a small portion of the MIMIC-IV dataset to fine-tune our model. Consequently, the GointMLP showed the best performance and achieved a positive $R^{2}$, indicating that the GointMLP can learn from a dataset with a completely different distribution using only a small amount of data. In particular, machine learning models, such as XGBoost, lack the capability for fine-tuning processes, highlighting the significant advantage of GointMLP.

GointMLP, as a multitask model, not only regresses vancomycin trough concentrations but also predicts vancomycin levels across three ranges. It performs these two tasks using a shared feature extractor, which enhances the model’s overall performance by extracting more generalized features and leveraging mutually beneficial features for each task. As shown in the results of Table 2, the output layer composed of regression combined with ordinal classification (S+J+D) demonstrated better performance compared to the output layer constructed with regression alone (S+J). This characteristic ensures that the predicted regression values and classification outputs are trained within the same context, significantly enhancing the model’s reliability through output comparisons. Notably, a match between the predicted vancomycin levels and the classifier’s ordinal prediction signals indicated high reliability, whereas a discordance between them suggested low confidence in the outputs. Unlike traditional regression AI systems, the proposed model supplies data to assess prediction reliability, enabling a system for informed clinical decisions.

Several factors must be considered when implementing our approach in a clinical setting. First, we utilized a larger dataset than previous studies, with 953 samples as the internal dataset and 1,379 samples as the external dataset. Furthermore, we employed the MIMIC-IV dataset of 2,557 samples to develop a generalized model. Consistent model performance is crucial for overcoming regional cultural differences and the varying proficiency levels of clinicians. Therefore, we evaluated the generalization performance of the proposed model using two datasets with varying data distributions: the Korean dataset and the MIMIC-IV dataset. Our results indicated that the proposed model fundamentally outperforms the other models and retains high performance across different data distributions, demonstrating superior generalization capability. Furthermore, our results demonstrate that the proposed model can achieve enhanced performance through fine-tuning with a small amount of data, suggesting its potential adaptability to other medical center datasets through similar fine-tuning processes. However, validation with external datasets remains crucial for enhancing our model’s generalizability in predicting vancomycin levels.

Despite incorporating patients from different regions, such as Asia, in the MIMIC-IV dataset to validate our model across diverse ethnicities, there is still room for enhancement through various external validations. To overcome the limitations of this retrospective study, we are currently conducting a clinical trial, aiming to validate further and confirm the efficacy of our product through DeepTDM. It focuses on assisting less experienced clinicians and maintaining the vancomycin trough level within the therapeutic range for critically ill patients by providing more accurate dosages and appropriate administration intervals. This work are expected to contribute to the development of artificial intelligence-based clinical decision support systems for critically ill patients, and particularly to advance the application of deep learning models in the field of personalized drug administration optimization.

We evaluated the predictive performance of our model against the PPK method using the trough-based target approach, which is more straightforward for treating critically ill patients compared to the currently recommended AUC/MIC-specific range. It is acknowledged that the importance of AUC/MIC and Bayesian models in pharmacokinetic analysis, which we recognize as the evolving standard of care in many international settings. While we are committed to aligning our research with these advancements, our current work deliberately focuses on a more pragmatic, translational approach tailored to the unique clinical environment of Korea. In Appendix, several practical constraints limit the widespread adoption of these methodologies. Nevertheless, given their current strong recommendation, future studies should aim to compare these methods with our method. Furthermore, socioeconomic status, which can significantly impact health outcomes and access to healthcare, was not considered in this study due to challenges in collecting these data. In addition, although dialysis status was included as a binary variable, neither the DeepTDM nor the PPK model incorporated any specific adjustments for dialysis patients. Consequently, prediction accuracy and applicability to this population may be limited and should be taken into account when interpreting our results. Future studies should consider including additional factors, such as socioeconomic status and dialysis status, which are known to influence patient outcomes. Our proposed model exhibits higher consistency in regression performance compared to the PPK method but tends to underestimate the prediction results. Notably, it yields larger errors for patients with vancomycin levels outside the 10- $20~\mu $g/mL range. This limitation arises because the data distribution is centered around the therapeutic range, which is the target for TDM treatment. Additionally, although the decision module enhances the model’s performance, there is a limitation where classification and regression results are inconsistent in some cases.

In future work, we plan to extend our model to incorporate AUC/MIC-based dosing, dialysis-specific adjustments, and broader patient factors such as socioeconomic status. We will also employ data augmentation strategies to mitigate underestimation for out-of-range cases and refine the decision module to reduce inconsistencies between regression and classification outputs. Despite these limitations, if our proposed model and product are applied in clinical settings, they can enhance treatment outcomes in intensive care units by providing more standardized and consistent support for clinical decision-making. The proposed GointMLP model and DeepTDM will be continuously refined and enhanced to mimic the human brain better and accurately determine the optimal vancomycin dose.

## VI. Conclusion

Introducing the GointMLP approach into clinical practice has substantial public health benefits, potentially optimizing treatment outcomes for patients in intensive care units. The model offers a more precise and consistent approach to predicting vancomycin levels, thereby reducing the adverse events associated with suboptimal dosing, including increased bacterial resistance, extended hospital stays, and higher healthcare costs. Furthermore, the GointMLP model enhances its performance through fine-tuning processes, resulting in more effective treatments and better patient health outcomes. This improvement can also enhance the performance of other clinical prediction models in the future. We have developed the program DeepTDM to enable our proposed model to be used effectively in real clinical settings, and we are currently conducting the regulatory approval process in the Republic of Korea to demonstrate its efficacy. If the clinical impact is ultimately proven through regulatory approval, implementing the GointMLP approach can significantly enhance public health outcomes and benefit patients worldwide.

## Appendix Constraints on AUC/MIC and Bayesian Model Use in Korean Clinics

We have recognized that AUC/MIC and Bayesian models are becoming the evolving standard of care in many international settings. While we are committed to aligning our research with these advancements, our current work deliberately focuses on a more pragmatic, translational approach tailored to the unique clinical environment of Korea. Here, several practical constraints limit the widespread adoption of these methodologies.

**High Program Costs:** Most commercially available Bayesian model-based dose optimization software requires expensive licensing fees. This poses a significant barrier for small- to medium-sized hospitals or regional medical institutions with limited budgets, making adoption challenging.
**Limited Expertise and Training:** Bayesian pharmacokinetic analysis demands specialized knowledge and extensive experience. In many hospitals with constrained staffing, implementing such complex processes routinely is often impractical.
**Data Accessibility Challenges** AUC/MIC-based monitoring relies on multiple time-point blood concentration measurements. In Korea’s healthcare system, factors such as patients’ financial burdens or difficulties with blood sampling frequently hinder the collection of sufficient data points. Moreover, considering the unique characteristics of ICU patients, who are the focus of this study, applying conventional methodologies consistently presents additional challenges.
**Dynamic Pharmacokinetic Changes:** ICU patients experience highly dynamic physiological changes, including hemodynamic instability, fluid volume fluctuations, and rapid variations in renal or hepatic function. In such cases, static pharmacokinetic models, including Bayesian approaches, struggle to deliver accurate predictions.
**Challenges in Real-Time Response:** Achieving AUC/MIC targets requires multiple drug concentration measurements, which becomes a practical burden in the fast-paced ICU environment where patient conditions change rapidly. Clinicians, tasked with managing critical parameters such as blood pressure, oxygen saturation, and electrolytes in real-time, face significant limitations in meticulously handling the complex pharmacokinetic metrics of individual antibiotics.

Our GointMLP model was designed to address these real-world ICU challenges.

**Real-Time Adaptive Prediction:** Leveraging GRU (Gated Recurrent Unit)-based time-series analysis, our model learns and adapts to patients’ dynamic physiological changes in real time. This enables it to capture subtle variations that static Bayesian models may overlook, facilitating more accurate and personalized dose predictions.
**Maximal Information from Minimal Data:** GointMLP delivers meaningful predictions using minimal data, such as trough concentrations. This reduces the burden of multiple blood samplings required for complex AUC/MIC calculations while still providing clinicians with reliable, real-time dosing recommendations.

Furthermore, the GointMLP model offers a practical solution to overcome the environmental constraints of Korea’s clinical settings.

## References

[1] M. J. Rybak , “Therapeutic monitoring of vancomycin for serious methicillin-resistant Staphylococcus aureus infections: A revised consensus guideline and review by the American society of health-system pharmacists, the infectious diseases society of America, the pediatric infectious diseases society, and the society of infectious diseases pharmacists,” Amer. J. Health-Syst. Pharmacy, vol. 77, no. 11, pp. 835–864, 2020. 10.1093/ajhp/zxaa036 32191793

[2] V. Bakke , “Vancomycin levels are frequently subtherapeutic in critically ill patients: A prospective observational study,” Acta Anaesthesiologica Scandinavica, vol. 61, no. 6, pp. 627–635, Jul. 2017. 28444760 10.1111/aas.12897 PMC5485054

[3] D. L. Burke, “Use of Bayesian methods for the design, analysis and synthesis of clinical trials,” Ph.D. dissertation, School Health Population Sci., Univ. Birmingham, Birmingham, U.K., 2015.

[4] T. Buclin, V. Gotta, A. Fuchs, N. Widmer, and J. Aronson, “An agenda for U.K. clinical pharmacology: Monitoring drug therapy,” Brit. J. Clin. Pharmacol., vol. 73, no. 6, pp. 917–923, Jun. 2012. [Online]. Available: https://onlinelibrary.wiley.com/doi/pdf/10.1111/j.1365-2125.2012.04237.x 22360377 10.1111/j.1365-2125.2012.04237.x PMC3391519

[5] D. Baracaldo-Santamaría, J. D. Cala-Garcia, G. J. Medina-Rincón, L. C. Rojas-Rodriguez, and C.-A. Calderon-Ospina, “Therapeutic drug monitoring of antifungal agents in critically ill patients: Is there a need for dose optimisation?,” Antibiotics, vol. 11, no. 5, p. 645, May 2022. 35625289 10.3390/antibiotics11050645 PMC9137962

[6] E. A. Poweleit, A. A. Vinks, and T. Mizuno, “Artificial intelligence and machine learning approaches to facilitate therapeutic drug management and model-informed precision dosing,” Therapeutic Drug Monitor., vol. 45, no. 2, pp. 143–150, Apr. 2023. 10.1097/FTD.0000000000001078 PMC10378651 36750470

[7] M. Bououda , “A machine learning approach to predict interdose vancomycin exposure,” Pharmaceutical Res., vol. 39, no. 4, pp. 721–731, Apr. 2022. 10.1007/s11095-022-03252-8 35411504

[8] X. Huang , “Prediction of vancomycin dose on high-dimensional data using machine learning techniques,” Expert Rev. Clin. Pharmacol., vol. 14, no. 6, pp. 761–771, Jun. 2021. 33835879 10.1080/17512433.2021.1911642

[9] S. Imai, Y. Takekuma, T. Miyai, and M. Sugawara, “A new algorithm optimized for initial dose settings of vancomycin using machine learning,” Biol. Pharmaceutical Bull., vol. 43, no. 1, pp. 188–193, 2020. 10.1248/bpb.b19-00729 31902925

[10] Z. C. Lipton, J. Berkowitz, and C. Elkan, “A critical review of recurrent neural networks for sequence learning,” 2015, arXiv:1506.00019.

[11] A. E. W. Johnson , “MIMIC-IV, a freely accessible electronic health record dataset,” Sci. Data, vol. 10, no. 1, p. 1, Jan. 2023. 36596836 10.1038/s41597-022-01899-x PMC9810617

[12] D. Kim , “A deep learning–based approach for prediction of vancomycin treatment monitoring: Retrospective study among patients with critical illness,” JMIR Formative Res., vol. 8, Mar. 2024, Art. no. e45202. 10.2196/45202 PMC10960205 38152042

[13] A. S. Levey , “Expressing the modification of diet in renal disease study equation for estimating glomerular filtration rate with standardized serum creatinine values,” Clin. Chem., vol. 53, no. 4, pp. 766–772, Apr. 2007. 17332152 10.1373/clinchem.2006.077180

[14] D. W. Cockcroft and H. Gault, “Prediction of creatinine clearance from serum creatinine,” Nephron, vol. 16, no. 1, pp. 31–41, 1976. 1244564 10.1159/000180580

[15] L. A. Bauer, Applied Clinical Pharmacokinetics, vol. 14. New York, NY, USA: McGraw-Hill, 2008.

[16] J. Chung, C. Gulcehre, K. Cho, and Y. Bengio, “Empirical evaluation of gated recurrent neural networks on sequence modeling,” 2014, arXiv:1412.3555.

[17] U. of California San Francisco. Vancomycin IV. Accessed: Oct. 25, 2025. [Online]. Available: https://idmp.ucsf.edu/content/vancomycin-iv

[18] J. F. Monteiro, S. R. Hahn, J. Gonçalves, and P. Fresco, “Vancomycin therapeutic drug monitoring and population pharmacokinetic models in special patient subpopulations,” Pharmacol. Res. Perspect., vol. 6, no. 4, p. 00420, Jul. 2018. 10.1002/prp2.420 PMC6113434 30156005

[19] T. Chen and C. Guestrin, “XGBoost: A scalable tree boosting system,” in Proc. 22nd ACM SIGKDD Int. Conf. Knowl. Discovery Data Mining, California, Aug. 2016, pp. 785–794.

[20] S. O. Arik and T. Pfister, “TabNet: Attentive interpretable tabular learning,” in Proc. AAAI Conf. Artif. Intell., vol. 35, 2021, pp. 6679–6687.

[21] W. Cao, V. Mirjalili, and S. Raschka, “Rank consistent ordinal regression for neural networks with application to age estimation,” Pattern Recognit. Lett., vol. 140, pp. 325–331, Dec. 2020.

[22] E. Štrumbelj and I. Kononenko, “Explaining prediction models and individual predictions with feature contributions,” Knowl. Inf. Syst., vol. 41, no. 3, pp. 647–665, Dec. 2014.

